# Spatial Heterogeneity of Household Food Consumption and Nutritional Characteristics of Grassland Transects in Inner Mongolia, China

**DOI:** 10.3389/fnut.2022.810485

**Published:** 2022-03-02

**Authors:** Wanni Yang, Haiwei Jia, Chao Wang, Haojia Wang, Chuanzhun Sun

**Affiliations:** ^1^China Center for Agricultural Policy, School of Advanced Agricultural Sciences, Peking University, Beijing, China; ^2^Institute of Geographic Sciences and Natural Resources Research, Chinese Academy of Sciences, Beijing, China; ^3^College of Resources and Environment, University of Chinese Academy of Sciences, Beijing, China; ^4^College of Public Management, South China Agricultural University, Guangzhou, China; ^5^Technical Centre for Soil, Agriculture and Rural Ecology and Environment, Ministry of Ecology and Environment of China, Beijing, China; ^6^College of Economics and Management, South China Agricultural University, Guangzhou, China

**Keywords:** food consumption patterns, food nutrition, grassland areas comparations, Inner Mongolia, Mongolian Plateau (MP)

## Abstract

Household food consumption is the associative link between ecosystems and anthropogenic activities. In grassland areas, inappropriate food consumption patterns will cause irreversible damage to vulnerable local ecosystems. For this study, we selected three typical transitional grassland areas of Inner Mongolia, China (i.e., meadow steppe, typical steppe, and desert steppe), to analyze spatial heterogeneity in household food consumption and nutritional characteristics. Results showed that: (a) Food consumption structures exhibited zonal gradients from east to west alongside a reduction in grassland conditions. Additionally, the average food consumption quantity also decreased. Available food supplies altered household preferences for vegetables and fruits, meat, dairy products, and other food consumption category types. (b) The nutritional structure implied that grains provided the main source of energy, proteins, and fat for local rural households, while meat, dairy products, beans (including bean byproducts), and oils caused a fluctuation in the nutritional structure of residents. (c) Local food supplies affect short-term local food consumption patterns, while socioeconomic development affects long-term food consumption patterns. This study is intended to provide a reference for the development of sustainable strategies for regional resource management.

## 1. Introduction

Food safety is critical for socioeconomic development, and it's also an important strategic basis for national development. While socioeconomic development and population growth have resulted in the diversified development of food consumption trends (1). Food security is also a key component of the 17 Sustainable Development Goals (SDGs) established by the 2015 United Nations General Assembly, and food consumption and nutrition are directly linked to SDG 2 (zero hunger) and SDG 12 (responsible consumption and production). However, the COVID-19 pandemic has undermined global food security. Consequently, nations globally have become increasingly concerned about national food system reliance (2–4). Much like the Government of China, many countries have shifted their attention and policy objectives to national food security (5, 6). The food consumption and nutritional structure of residents will therefore have an impact on the food system and food security.

Food consumption concerns ecosystem and socioeconomic system research (7). Moreover, various socioeconomic development constructs will alter different regional food consumption and nutritional patterns differently (8, 9), and such changes will have an impact on the physical and mental health of consumers while also impacting local ecosystems and climate change conditions (10–12).

Among the various ecosystem types, grassland is particularly vulnerable (13). For example, inappropriate anthropogenic activities (i.e., overgrazing, excessive reclamation, etc.) will negatively affect local ecosystems, and many resultant effects will either be irreversible or necessitate considerable restoration costs (12, 14). Moreover, people who reside in grassland areas rely heavily on them; thus, their livelihoods and local environments are intrinsically inseparable (15, 16). However, the food that grassland ecosystems provide is limited, particularly under crises, such as strong storm events, the COVID-19 pandemic, etc., and the potential food supplies which can be produced are subsequently finite (17–19). These are the challenges that grassland areas in China face, especially in Inner Mongolia, being one of the largest grassland areas in China.

Food consumption studies conducted in Inner Mongolia have mainly focused on food consumption demands, food supplies, local food productivity, and local sustainability (20–22). As local rural residents shifted from nomadism to set stocking (i.e., grazing livestock in paddocks), food consumption practices and the nutritional structure of local residents have changed significantly (23–25). The main change observed in consumption patterns was from plant-based to animal-based food sources (9, 26). Although this has led to diversity in local food consumption to some extent, it has also led to the overconsumption of animal-based food products, which has subsequently resulted in health problems (27, 28).

Despite the implementation of the Grassland Ecological Compensation Policy (GECP), rural resident livelihoods in China face many other challenges (29). The food consumption impact of grassland areas is complex (6, 17). To add to this complexity, Inner Mongolian grassland area is spatially heterogeneous easterly to westerly. Understanding the spatial and temporal distribution of vegetation can provide a scientific basis for evaluating its regulating effect on climate and provide decision support for formulating adaptive management strategies of the correspondence ecosystems (30, 31). Although studies have been conducted on changes in Inner Mongolian food consumption patterns in recent years, there have been relatively few food consumption studies from the perspective of regional heterogeneity, and studies on food consumption drivers require more short-term and long-term analysis. This study endeavored to analyze characteristics of food consumption and nutrition patterns among the different grassland transitional zones of Inner Mongolia to fill the research gaps. For this investigation, we selected Hulun Buir, Xilin Gol, and Ordos as the study sites, which represent typical meadow steppe, typical steppe, and desert steppe ecoregions. The objectives of this study were as follows: (1) to analyze food consumption patterns and the nutritional structure of household residents residing in these three transitional grassland areas; (2) to reveal spatiotemporal characteristic changes within these three different grassland land-use types; (3) to explore food consumption drivers of local residents from a spatial perspective. This study is intended to enhance our understanding of food consumption structure characteristics and subsequent impacts to grassland areas in Inner Mongolia. It also provides a scientific reference to strengthen the resilience on our reliance of food systems while realizing the sustainable development of regional resources and the environment.

## 2. Methods

### 2.1. Study Areas

Inner Mongolia is situated in northern China. It belongs to the Mongolian Plateau, in which China's temperate grasslands are mainly distributed, with an average elevation of 1,000 m.a.s.l (32). The total area of Inner Mongolia is 1.2 × 10^6^ km^2^, and it is the third largest administrative division (i.e., autonomous region) in China. Inner Mongolia is under the influence of a temperate continental monsoon climate, and it experiences low and uneven precipitation as well as dramatically shifting temperatures. Annual precipitation ranges from 50 to 500 mm, and annual pan evaporation ranges between 1,000 and 3,000 mm. The winter season is long and cold, with an average monthly temperature of 10–32°C in January. The summer season is warm and short, with an average monthly temperature range from 16 to 27°C in July. The region is comprised of nine cities and three leagues (where a league is defined as a city-level administrative division of Inner Mongolia). At the conclusion of 2019, the total registered population of the region was 2.5 × 10^7^, of which rural people accounted for 36.6%. The ancestry of the population is 76.9% Han Chinese and 19.2% Mongolian. In 2019, the per capita disposable income of residents was 30,555 yuan per year, of which the per capita disposable income of urban residents was 40,782 yuan per year vs. 15,283 yuan per year for rural residents. The GDP was 1.7 × 10 ^13^ yuan, and the per capita GDP was 67,852 yuan in 2019.

**Figure 1:**
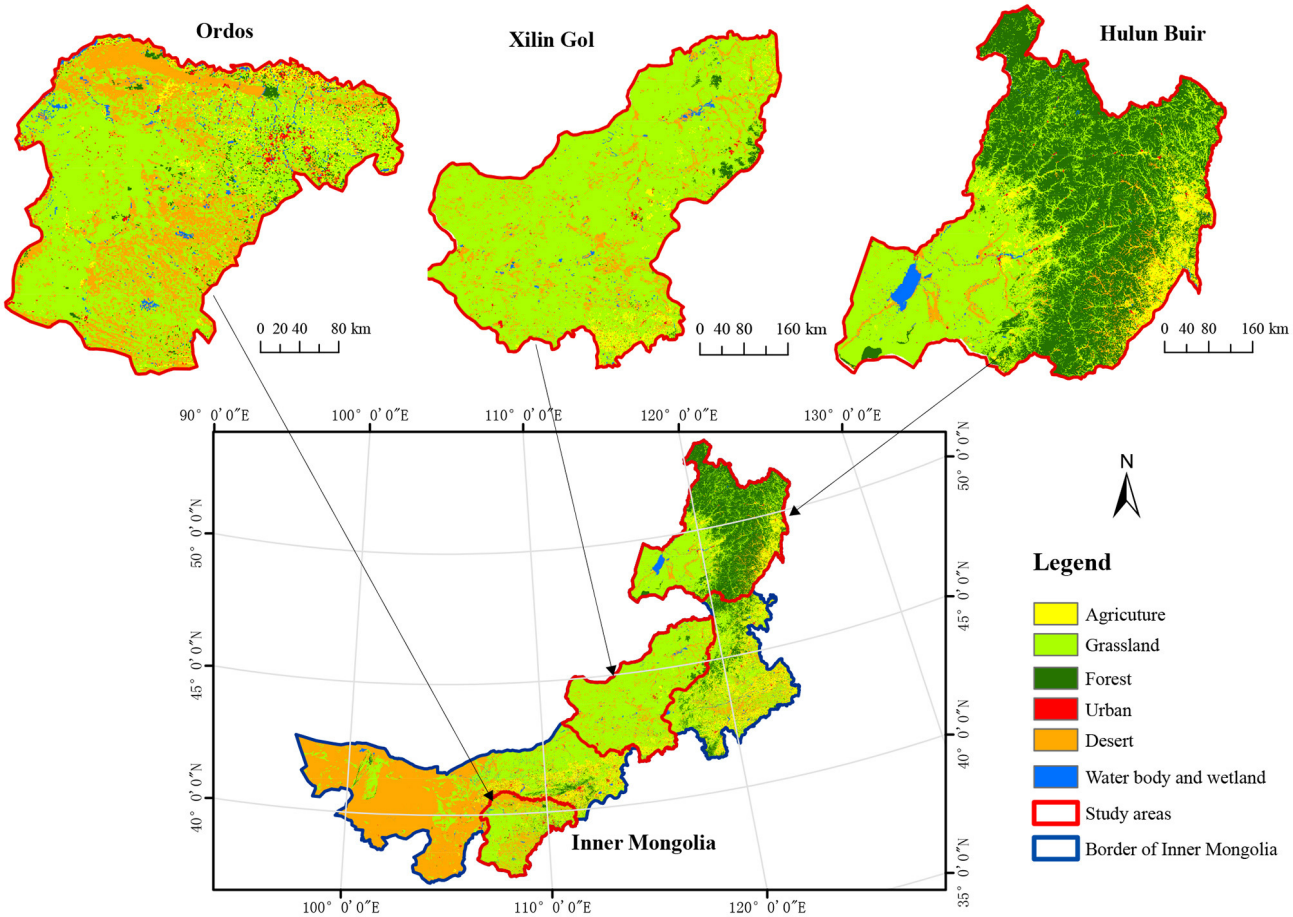
Location of study sites in Inner Mongolia and the dominant land-use types. Source: data center for Resource and Environment Data Platform of the Chinese Academy of Sciences (CAS) (http://www.resdc.cn).

The climate of the region is zonally distributed, consisting of an arid to semi-arid continental climate with strong climatic gradients, grassland being the dominant land-use type (Figure 1). Annual average temperatures in this region vary from 5 to 10°C, and annual precipitation ranges from 35 to 530 mm (33, 34). Additionally, the region consists of three main grassland types: meadow steppe, typical steppe, and desert steppe. For this study, three areas of Inner Mongolia were selected as city-level study sites: Hulun Bair, Xilin Gol, and Ordos. These city-level areas exemplify three typical and fragile grassland zones which are themselves representative of typical grassland zones found in Inner Mongolia. Basic information on these three cities is provided in Table 1. Among these three cities, Hulun Buir has the largest overall land area (2.5 × 10^5^ km^2^), located in the northeast of the region. Xilin Gol is centrally located with a land area of 2.0 × 10^5^ km^2^. Ordos is located in the southwest of the region and has the smallest overall land area (8.7 × 10^4^ km^2^), located near to the Alashan Plateau semi-desert.

**Table 1:** Basic information about the study areas (2012).

**Study area**	**Grassland type**	**Land area** **(×10^**4**^** **km^**2**^)**	**Total resident** **population** **(×10^**4**^)**	**Population** **Mongolian** **(%)**	**Urbanization** **(%)**	**GDP** **(×10^**9**^ yuan)**	**GDP per** **capita (yuan)**	**Average income** **of rural residents** **in 2012 (yuan)**
Hulun Buir	Meadow steppe	25.3	253.4	9.1	69.0	1335.8	52,649	8,807
Xilin Gol	Typical steppe	20.3	102.0	30.8	45.6	820.2	79,105	8,925
Ordos	Desert steppe	8.7	104.1	12.8	31.6	3656.8	182,680	11,416

*Source: statistical yearbooks of Hulun Buir, Xilin Gol, and Ordos (35–37)*.

### 2.2. Data Collection

This study mainly used various types of data, including statistical data on household resident food consumption patterns of the selected study sites, statistical data on the food nutrition content of Chinese residents, data on land-use types, and the socioeconomic data of residents (i.e., income, consumption expenditure, agricultural and livestock product output, per capita grassland area, etc.).

First, the resident food consumption data (2000–2012) were obtained from statistical yearbooks issued by the Bureau of Statistics of Hulun Buir, Xilin Gol, and Ordos, respectively, all of which were compiled from published yearbooks obtained from the National Library of China. Then, the source information (derived from the municipal websites of the three selected sites) was used as supplementary sources for certain indicators. Since the statistical yearbook data derived from household sample surveys were taken in pastoral areas by survey teams of the statistical bureaus of the various cities, results of the data represent the real-life conditions of pastoral residents at the sites selected for this study (Table 2).

**Table 2:** Data and sources used in this study.

**Data**	**Data content and application**	**Source**
Food consumption data of rural residents	Food consumption quantity and structure of rural residents	(1) Statistical yearbook of Hulun Buir (35); (2) Statistical yearbook of Xilin Gol (36); (3) Statistical yearbook of Ordos (37);
Land use data	Study area location and land use data	(1) Data Center for Resources and Environmental Sciences, Chinese Academy of Sciences (1 × 1 km, http://www.resdc.cn/, 2015) (2) European Space Agency (ESA), (300 × 300 m, http://maps.elie.ucl.ac.be/CCI/viewer/download.php, 2000–2012)
Food nutrition data	Data on energy, fat, and protein content of each food type in China	China Food Composition (38)
Ecological and socio-economic characteristic data of the study sites	Indicators such as NPP, NDVI, grassland type, grassland area, residenthousehold rearing structure, household income, ethnic population composition, family size, regional transportation facilities, residential housing, etc.	(1) NPP and NDVI data obtain from Data Center for Resources and Environmental Sciences, Chinese Academy of Sciences (1 × 1 km, http://www.resdc.cn/, 2000–2012) (2) Statistical yearbook of Hulun Buir (35); (3) Statistical yearbook of Xilin Gol (36); (4) Statistical yearbook of Ordos (37); (5) Official government websites of Hulun Buir (http://hlbe.gov.cn), Xilin Gol (http://www.xlgl.gov.cn/) and Ordos (http://ordos.gov.cn/).
Food consumption and nutritional data of typical grassland countries	Food consumption quantity and nutritional data on food comprised of fat, protein, and energy	FAO database (http://www.fao.org/faostat/en/#data)

For spatial data, the data used in this study include study site locations, land-use type data, the net primary production (NPP) and normalized difference vegetation index (NDVI) data. These data sources are based on Landsat eight remote sensing images and generated using manual visual interpretation (Table 2). The land-use type data of 1 × 1 km were obtained from the Data Center for Resources and Environmental Sciences, the Chinese Academy of Sciences (CAS) (http://www.resdc.cn/). Land-use types were divided into six primary groups, including water bodies and wetlands, grassland, agricultural land, forests, urban areas, and desert areas, according to the National Land Survey (NLS) of China (2002 standard). Land use data from European Space Agency (ESA) with more classification information and continuous years was used to analyze land use change impact on food consumption (300 × 300 m, http://maps.elie.ucl.ac.be/CCI/viewer/download.php). Moreover, annual NPP and NDVI data from 2000 to 2012 were obtained from the Data Center for Resources and Environmental Sciences, the Chinese Academy of Sciences (CAS) (1 × 1 km, http://www.resdc.cn/).

### 2.3. Data Analysis Methods

#### 2.3.1. Cataloging and Division of Food Consumption Categories

According to the local statistical yearbooks, we divided all the food consumed by local residents at the study sites into 11 categories and 36 sub-categories for statistical analysis. In view of the structure of the different food type consumption patterns in the grassland areas selected for this study (i.e., where people showed a preference for meat, milk, and dairy), food types were assorted into five categories for analysis: (1) grains, (2) vegetables and fruits, (3) meats, (4) eggs and milk, (5) and others. The category “others” includes oil, beans and bean byproducts, tea, sugar, and alcohol (Table 3).

**Table 3:** The main food types and items consumed by rural residents at the study sites.

**Food types and food items**		
**Grains**	**Meat**	**Dairy**
Wheat	Pork	Milk
Rice	Beef	Dried milk cake
Corn	Mutton	Other dairy products
Broomcorn	Poultry	**Alcohol**
Millet	Meat and poultry products (MPP)	Liquor and spirits
Other cereals	Fish	Beer
Sweet potato	**Vegetable**	Fruit wine
Potato	Root tubers	**Oil**
Other yams	Snake melon (i.e., Armenian cucumber)	Vegetable oil
**Eggs**	Nightshade	Animal oil
Chicken eggs	Cabbage	**Beans and bean byproducts**
Duck eggs	Leafy greens	Soybean
Other eggs	Other fresh vegetables	Other beans
**Fruits**	Dried vegetables	Soy products
Melon		**Others**
Fruit		Tea
		Sugar

#### 2.3.2. Conversion Method and Standard of Food Nutrition

For this study, we analyzed the consumption and nutritional structure of food consumed by rural residents of Inner Mongolia under the framework of the Dietary Guidelines for Chinese Residents released by the Chinese Nutrition Society. Referring to the Chinese Dietary Guidelines 2016 (CDG-2016), we mainly analyzed the status of and the changes to food consumption characteristics and the associated nutritional structure between 2000 and 2012. We mostly focused on indexes related to food consumption quantity, energy, protein, and fat. Table 4 provides the nutritional content of the main food types consumed by rural residents at the study sites. Using food consumption data from these sites, we calculated the daily per capita consumption of all food types at the study sites and referred to the nutritional content table to analyze the average daily food intake per individual, which provided us data on annual food intake and nutrient intake from 2000 to 2012.

**Table 4:** Nutritional content of food types (content per 100 g of edible food).

**Food type**	**Energy** **(kcal)**	**Protein** **(g)**	**Fat** **(g)**	**Food type**	**Energy** **(kcal)**	**Protein** **(g)**	**Fat** **(g)**
**Grains**				**Fruits**			
Wheat	339.0	11.9	1.3	Melon	27.5	0.5	0.1
Rice	347.0	7.4	0.8	Fruit	94.9	0.9	0.2
Corn	112.0	4.0	1.2	**Vegetable**			
Broomcorn	360	10.4	3.1	Root tubers	36.9	3.3	0.2
Millet	361	9	3.1	Snake melon	311.6	12.5	4.6
Other cereals	376	12.2	7.2	Nightshade	49.5	7.3	0.3
Sweet potato	102	1.1	0.2	Cabbage	153.2	13.1	1.7
Potato	77	2	0.2	Leafy greens	277.0	20.8	2.5
Other yams	119	2.1	0.3	Other fresh vegetables	23.5	0.5	0.1
**Eggs**				Dried vegetable	36.9	3.3	0.2
Chicken eggs	144.0	13.3	8.8	**Dairy**			
Duck eggs	180.0	12.6	13.0	Milk	54.0	3.0	3.2
Other eggs	162	10.9	12.95	Dried milk cake	305.0	46.2	7.8
**Meat**				Other dairy products	235.7	19.3	5.4
Pork	395.0	13.2	37.0	**Alcohol**			
Beef	125.0	19.9	4.2	Liquor and spirits	351.0	0.0	0.0
Mutton	203.0	19.0	14.1	Beer	32.0	0.4	0.0
Poultry	203.5	17.4	14.6	Fruit wine	72.0	0.1	0.1
Meat and poultry products	231.6	17.4	17.5	**Oil**			
Fish	103.0	16.6	3.3	Vegetable oil	898.0	0.0	99.7
**Beans and bean byproducts**				Animal oil	828.7	0.0	89.57
Soybean	390.0	35.0	16.0	**Others**			
Other beans	365.5	29.0	8.2	Sugar	400.0	0.0	0.0
Bean byproducts	189.3	19.2	8.7	Tea	283.0	14.5	4.0

*Source: China Food Composition (38)*.

The formula used to calculate food nutrition corresponding to food consumption is as follows:


$$\begin{array}{l} N_{j}=\sum F_{ij}\times n_{ij} \end{array}$$

where i is the food category, which includes the 40 categories of food consumed at the study sites (i.e., grains, eggs, meats, beans and bean byproducts, fruits, vegetables, dairy, etc.); j is the nutritional category, which includes energy, protein, and fat; F is the food consumption quantity; n is the food nutrient content.

## 3. Results

### 3.1. Food Consumption Characteristics

**Figure 2:**
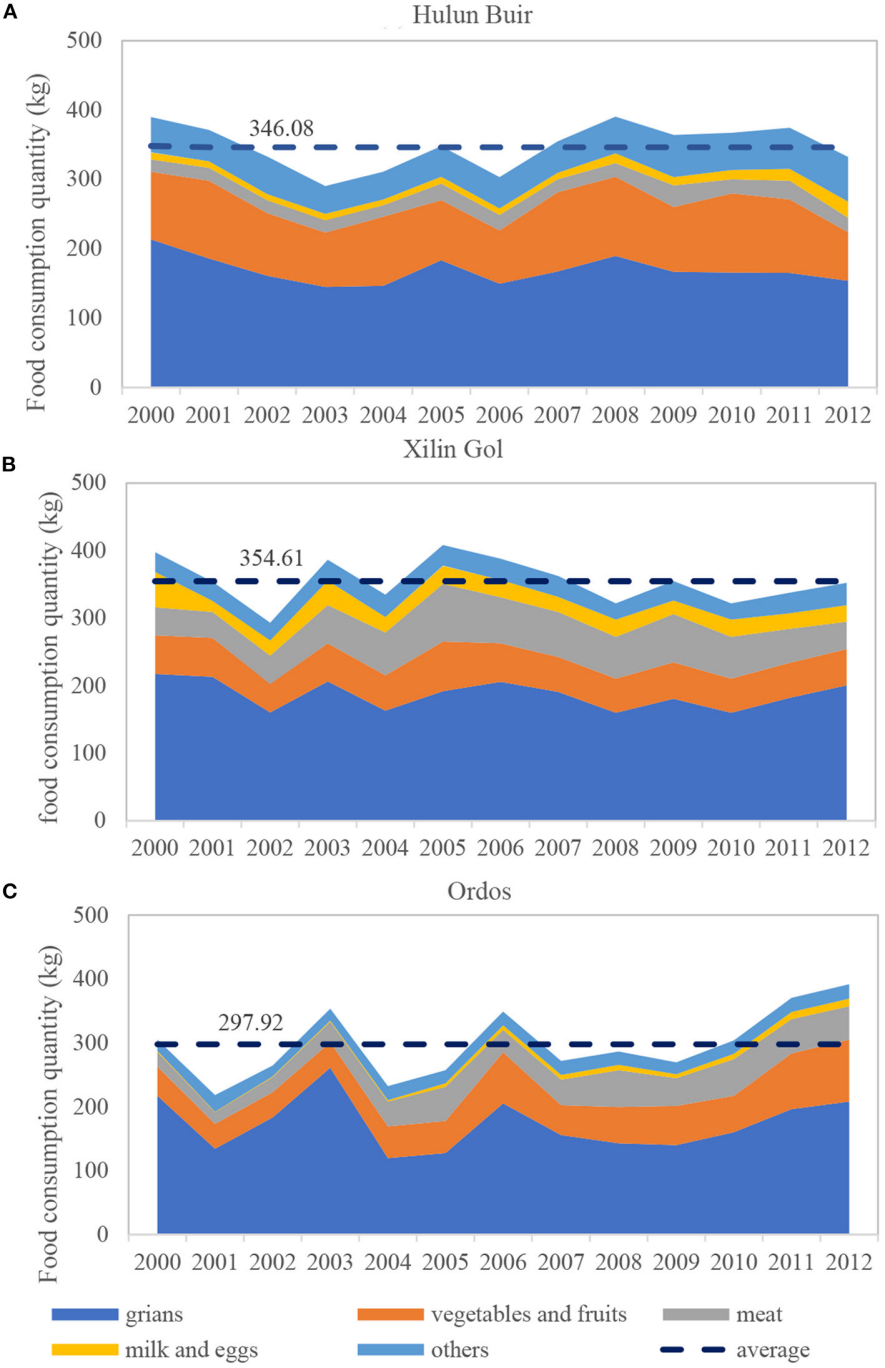
The food consumption structure of residents (2000–2012). Data source: Statistical yearbooks from 2000 to 2012 that were assorted and calculated by the authors of this study. **(A)** Hulun Buir, **(B)** Xilin Gol, **(C)** Ordos.

Through an analysis of food consumption quantities and structures in Hulun Buir, Xilin Gol, and Ordos between 2000 and 2012, we found a zonal gradient (from east to west) in the food consumption pattern of rural residents. From east to west, the food consumption quantity (from the perspective of the total per capita food consumed by residents) decreased; namely, the per capita average annual food consumed in Hulun Buir (346.08 kg) and Xilin Gol (354.61 kg) was higher than in Ordos (297.92 kg) in the south (Figure 2).

Food consumption structures showed spatial heterogeneity. Grains were the most important food category consumed by rural residents at all study sites (>50%). Meanwhile, the annual per capita consumption of vegetables and fruits exhibited a gradual decreasing trend from east to west (i.e., vegetable consumption quantity: 80.61 kg in Hulun Buir, 45.57 kg in Xilin Gol, and 42.59 kg in Ordos; fruit consumption quantity: 15.79 kg in Hulun Buir, 9.05 kg in Xilin Gol, and 15.19 kg in Ordos). However, changes in the quantity of meat consumption differed in that residents in the southern desert steppe (Ordos, 40.07 kg) consumed more food than residents in the typical steppe (Xilin Gol, 28.14 kg) and meadow steppe (Hulun Buir, 18.03 kg). Specifically, residents in rural Xilin Gol consumed more eggs and milk compared to the other two study sites, showing a stronger preference for these food products (Figure 2 and Table 5).

**Table 5:** Quantities of annual food types consumed by rural residents at all three study sites and one-way ANOVA test results.

**Factor**	**One-way ANOVA**		**Hulun Buir**		**Xilin Gol**		**Ordos**	
	***F***	***p*-value**	**Mean (kg)**	**Variance**	**Mean (kg)**	**Variance**	**Mean (kg)**	**Variance**
**Grains**	1.71	0.195^*ns*^	164.80	574.16	186.77	445.80	173.00	1794.52
**Vegetables and fruits**								
Vegetables	46.11	<0.001^***^	80.61^*a*^	234.65	45.57^*b*^	58.68	42.59^*b*^	84.82
Fruits	3.66	0.036^*^	15.79^*a*^	6.93	9.05^*b*^	4.72	15.19^*a*^	136.29
**Meats**								
Meat	19.26	<0.001^***^	18.03^*c*^	15.75	28.14^*b*^	46.80	40.07^*a*^	182.09
Fish	236.21	<0.001^***^	3.01^*a*^	0.25	0.63^*b*^	0.02	0.60^*b*^	0.04
**Dairy and egg**								
Milk & Dairy	51.93	<0.001^***^	6.36^*b*^	14.48	25.00^*a*^	76.94	3.98^*b*^	7.71
Eggs	84.12	<0.001^***^	5.93^*a*^	1.08	1.61^*b*^	0.17	1.73^*b*^	1.57
**Others**								
Alcohol	40.29	<0.001^***^	15.85^*a*^	15.76	10.77^*b*^	1.98	6.75^*c*^	2.37
Tea	3.64	0.036^*^	1.45^*a*^	1.93	4.42^*a*^	42.67	0.48^*b*^	0.65
Bean and bean byproducts	8.98	0.001^**^	9.22^*a*^	74.21	1.24^*b*^	1.13	2.94^*b*^	1.28
Oil	31.12	<0.001^***^	6.89^*a*^	4.35	2.11^*b*^	0.78	2.84^*b*^	3.16
Sugar	7.81	0.002^**^	0.89^*b*^	0.01	1.39^*a*^	0.07	1.04^*b*^	0.25

*Significance (one way ANOVA): ns, not significant*;

* *p < 0.05*,

** *p < 0.01, and*

*** *p < 0.001. “a,” “b,” and “c” show the significant difference among study sites at 5% level*.

One-way analysis of variance (one-way ANOVA) was used to analyze the food consumption characteristics of rural residents at all three study sites. Results showed that in addition to grain, both the amount and structure of the other food categories consumed by residents differed significantly (*p* < 0.01) between the study sites (Table 5).

Regarding the food categories, the consumption of vegetables, meat, eggs, milk, and other food types differed significantly among the three sites. Specifically, vegetable consumption gradually decreased from east to west, and the consumption of vegetables in Hulun Buir in the east was almost twice that of residents in Ordos in the south. However, differences in fruit consumption were not significant (Table 5).

Meat consumption differed among the three sites. With regard to quantity, the meat consumption of residents increased from east to west. From a structural point of view, pork was the dominant meat type consumed by residents of Hulun Buir. Mutton was the dominant meat type consumed by residents of Xilin Gol, supplemented by beef and pork, and this was because this region is known as one of China's largest sheep breeding areas, which subsequently encourages mutton consumption. Pork was the dominant meat type consumed by residents of Ordos, supplemented by mutton (Table 6). Additionally, fish consumption in Hulun Buir was greater than that of the other two sites, and this is due to the expansion of Lake Hulun in combination with an increase in local food supplies. Besides, regarding the consumption of eggs and milk, egg consumption decreased from east to west while milk consumption in Xilin Gol was especially high (Table 5).

**Table 6:** Meat consumed by residents at all three study sites (2000–2012 averages).

**Factor**		**One-way ANOVA**		**Hulun Buir**		**Xilin Gol**		**Ordos**	
		***F***	***p*-value**	**Mean (kg)**	**Variance**	**Mean (kg)**	**Variance**	**Mean (kg)**	**Variance**
Mutton		35.54	<0.001^***^	1.62^*b*^	0.92	13.33^*a*^	13.63	12.60^*a*^	34.09
Beef		18.79	<0.001^***^	1.90^*c*^	1.62	5.66^*a*^	2.43	3.21^*b*^	3.52
Chicken		9.72	0.001^**^	2.23^*a*^	0.84	0.77^*b*^	0.13	2.29^*a*^	2.18
Pork		40.65	<0.001^***^	12.20^*b*^	4.42	8.36^*c*^	10.44	21.97^*a*^	32.35
Fish		236.21	<0.001^***^	3.01^*a*^	0.25	0.63^*b*^	0.02	0.60^*b*^	0.04

*Significance (ANOVA): ns, not significant*;

** *p < 0.01*;

*** *p < 0.001. “a,” “b,” and “c” show the significant difference among study sites at 5% level*.

In these transitional grassland areas, we found that food consumption structures fluctuated and changed over time. The food consumption quantity of eggs and milk increased in Hulun Buir and Ordos between 2000 and 2012 (Figure 2). Moreover, increases in the consumption of vegetables and fruits in Ordos was due to construction and infrastructure developments, such as highways and means of delivery. Although grain consumption quantities did not change significantly in these areas, grain consumption decreased proportionally, showing that resident consumption behavior in these three grassland areas reflected changes in food consumption structure (9, 26); The fluctuations of grains were shown in three grassland areas, which were due to the fluctuations of corn's consumption quantity. The consumption quantity of corn were fluctuated among the years, and the reasons of fluctuations of corn consumption quantity need further analysis in the future; Additionally, fluctuations in food consumption quantities were also due to changes in the definition of the food categories. For example, the quantity of grain consumed in Ordos fluctuated significantly in 2006, which was due to a change in the definition of “other yams” in 2006, where a new type of yam was introduced, subsequently increasing the consumption quantity of this category.

### 3.2. Food Nutritional Characteristics

Nutritional food structures among these three transitional grassland areas only showed differences in protein and fat intake. In Hulun Buir and Xilin Gol, the intake of energy and proteins was higher compared to Ordos. Moreover, the food consumption quantity in Hulun Buir and Xilin Gol was higher compared to Ordos (Figure 2 and Table 5). Local residents from Hulun Buir and Xilin Gol tend to consume more foods such as beans (and bean byproducts), oil, and sugar, which subsequently contribute to their overall protein and fat consumption.

**Figure 3:**
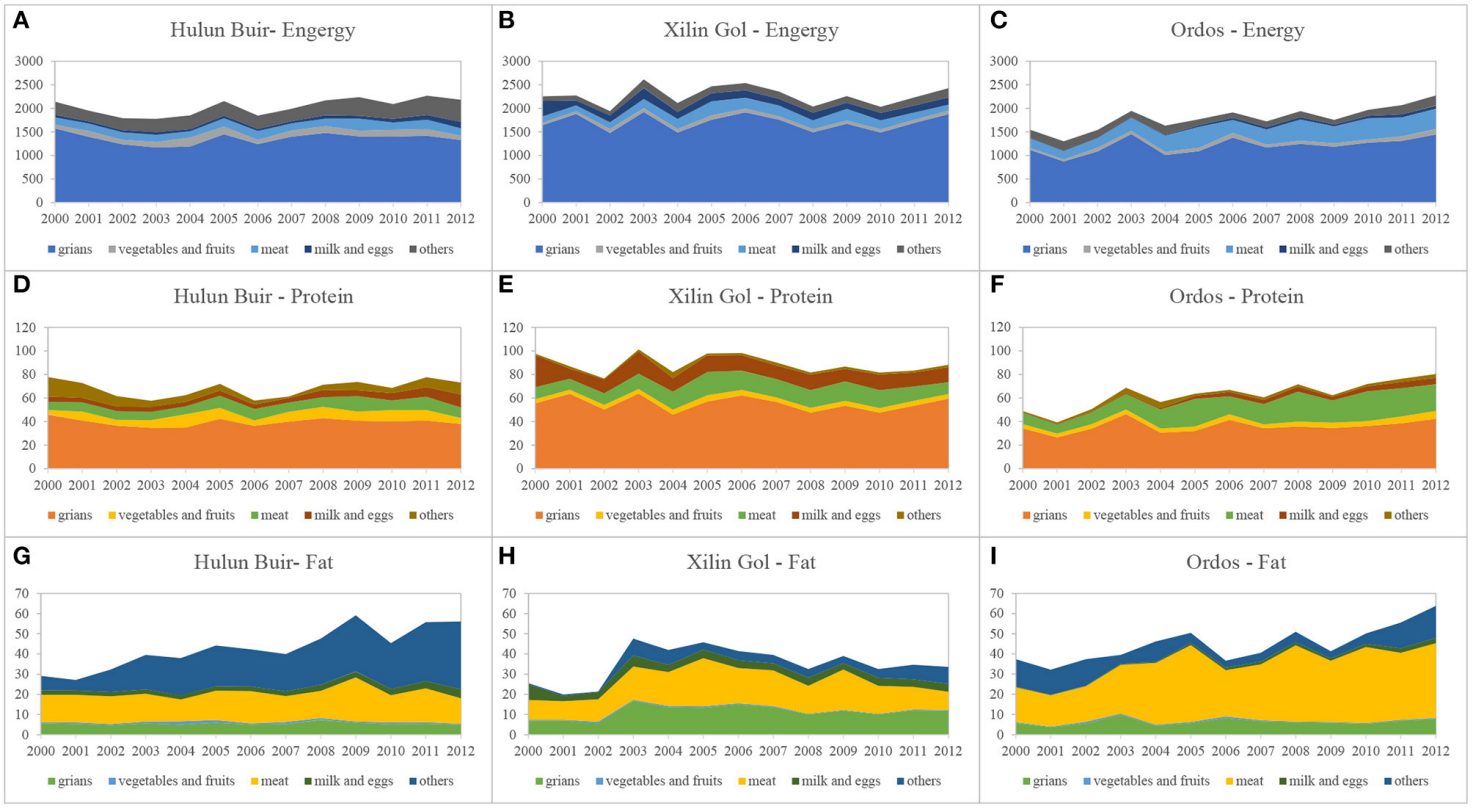
Nutritional characteristics of rural residents in three study sites (2000–2012). **(A–C)** Energy intake of rural residents. **(D–F)** Protein intake of rural residents. **(G–I)** Fat intake of rural residents. Data source: calculated by the authors.

Interannual changes in the energy intake of residents of these three grassland areas were negligible (Figures 3A–C). Xilin Gol residents had the highest overall calorie intake, followed by Hulun Buir and Ordos with the least. Grains provided the main source of energy, while there was a proportional and gradual increase in meat throughout the study years.

Protein intake in Xilin Gol was relatively high and stable, while it fluctuated significantly in Hulun Buir and rose only slowly in Ordos (Figures 3D–F). Among these Inner Mongolian grassland areas, we observed structural differences among the main protein sources. Grains were the main protein source in Hulun Buir, while other food categories fluctuated significantly, having a greater impact on the total amount. Grains, eggs, milk, and meat were the main protein sources for residents of Xilin Gol, and the structure was determined to be stable. Grains were the main protein source in Ordos (followed by meat), while an abnormal value was observed in 2011, which resulted from the unique grain consumption structure of that particular year.

The overall fat intake of residents was relatively stable as was the proportion of fat sources (Figures 3G–I). Meat and “others” (mainly oil) were the main fat sources for residents of Hulun Buir. Meat and grains were the main fat sources for residents of Xilin Gol. Finally, meat was the dominant fat source for residents of Ordos.

Fluctuations of nutrients were also shown among three grassland areas. In Hulun Buir, fluctuations in protein and fat intake were due to fluctuations in other food categories. Moreover, consumption quantities of protein-rich beans (and bean byproducts) and fat-rich oil in Hulun Buir were highest overall among these three transitional grassland areas. The high intake of these protein-rich and fat-rich food categories resulted in the high protein and fat intake of Hulun Buir (Figure 3). Additionally, fluctuations in fat were mainly due to changes in the definition of the food categories. In Xilin Gol, “other yams” weren't included in the grains in 2000–2002, which were fat-rich food. The leakage of “other yams” led to the underestimation of fat intake in Xilin Gol in 2000–2003 (Figure 3).

## 4. Discussions

### 4.1. Effects of Ecological Factors and Associative Spatial Heterogeneity

Spatial heterogeneity of food consumption changed alongside the regional ecological status, which also showed spatial heterogeneity. The heterogeneity of ecological factors in grassland transects may affect local rural household food consumption and nutritional patterns by affecting food supplies from land use and land productivity.

The ecosystems and land use of these grassland areas are relatively unique and stable, which simplifies food system supplies (22). On one hand, grassland and cropland areas, which are the major land types that produce food productions in grassland areas, showed little change in our study sites during 2000–2012. As Table 7 shows, the covariance of grassland (0.004, 0.006, 0.016) and cropland (0.003, 0.017, 0.007) in Hulun Buir, Xilingol, and Ordos were extremely small from 2000 to 2012 (covariance <1). These indicate that the stable land use ensured stable productivity in these areas. On the other hand, grassland and cropland areas were significantly different within the three grassland transects. The land-use diversity among the three grassland areas reveals the spatial heterogeneity in the livelihoods of rural residents, which affected local food supplies. Table 8 shows the pastureland status of the three sites in 2012. In Hulun Buir, grassland comprised 38.3% of all land-use types, where pastureland accounted for ~813,374 km^2^, while pastureland per capita was 1.03 km^2^, for which was the highest among the three grassland sites. This was followed by Xilin Gol and Ordos. However, 81.8% of land-use area in Xilin Gol is grassland, and its typical steppe is more suitable for grazing (i.e., Xilin Gol grassland is considered one of the five best pastureland areas in China). Thus, local resources support raising more animals (especially sheep). Differences in supply also reflected the food consumption structure of household residents, and rural residents in Xilingol tend to consume more mutton compared to the other two sites, but dairy consumption is relatively lower (Figure 2 and Table 6). Other relevant studies conducted in Inner Mongolia (39–41) as well as other grassland areas (42–44) reported similar findings.

**Table 7:** NPP, NDVI, grassland, and cropland areas of the three study sites (2000–2012 averages).

**Factor**	**one-way ANOVA**		**Hulun Buir**		**Xilin Gol**		**Ordos**	
	***F***	***p*-value**	**Mean**	**Variance**	**Mean**	**Variance**	**Mean**	**Variance**
NPP (gC/m^2^yr)	177.85	<0.001^***^	299.16^*a*^	791.54	191.44^*b*^	374.51	129.96^*c*^	318.68
NDVI	475.37	<0.001^***^	0.2608^*a*^	0.0002	0.1689^*b*^	<0.0001	0.1391^*c*^	<0.0001
Grassland areas (km^2^)	16618.27	<0.001^***^	84855.79^*b*^	9741.12	135534.62^*a*^	681132.42	77177.24^*c*^	1581128.34
Cropland areas (km^2^)	934392.38	<0.001^***^	32017.73 ^*c*^	6498.76	3767.74 ^*a*^	4265.50	3390.95 ^*b*^	489.09

*Significance (one-way ANOVA): ns, not significant*;

*** *p < 0.001. “a,” “b,” and “c” show the significant difference among study sites at 5% level*.

*Sources: European Space Agency (http://maps.elie.ucl.ac.be/CCI/viewer/download.php); Data Center for Resources and Environmental Sciences, the Chinese Academy of Sciences (CAS) (http://www.resdc.cn/)*.

**Table 8:** Production patterns of rural residents under existent natural resources (2012).

	**Hulun Buir** **(Meddow Steppe)**	**Xilin Gol** **(Typical Steppe)**	**Ordos** **(Desert Steppe)**
% of grassland area	38.3	81.8	59.7
**Pasture status**			
Total pasture area (km^2^)	813,374	176,556	58,264
Pasture area per capita	1.03	0.32	0.06
(km^2^)			
**Breeding structure (per capita average)**			
Sheep	2.35	4.85	1.69
Cattle	0.40	0.80	0.14
Horse	0.06	0.10	0.01
Pig	0.14	0.05	0.28

*Grassland % data were calculated from 2012 land-use data*.

*Sources: European Space Agency (http://maps.elie.ucl.ac.be/CCI/viewer/download.php); statistical yearbooks of Inner Mongolia and local government websites of Hulun Buir, Xilin Gol, and Ordos*.

Along with the spatial heterogeneity in land use, land productivity would also affect the local food production supplies. NPP and NDVI are widely used indicators for estimating the ecological status of grassland areas (25), which could better reflect the quality of vegetation among different grassland areas. In our study sites, we found that: first, both NPP and NDVI showed spatial heterogeneity in all three grassland areas. As Table 7 shows, the NPP and NDVI of all three sites throughout the study period (2000–2012) significantly differed (*p* < 0.001), which suggests the existence of natural regional resources even though all three sites were grassland. NPP and NDVI were highest in Hulun Buir followed by Xilin Gol and Ordos (Table 7). Factors affect NPP and NDVI among different areas in Inner Mongolia includes temperature, land-use changes, urbanization, and other human activities (30, 33). Second, taking NPP as an example to explore the relationship between ecological factors and food consumption, regression results showed that NPP affects food consumption. Especially, NPP significantly positively affected the consumption of fruits, chicken, fish and eggs (Table 9), which implies that better NPP would promote rural residents' consumption of high protein and rich micro-nutrients, and these food items were consumed less by residents in our study sites compared to the CDG-2016 recommendations. Finally, previous studies also similarly found that NPP or NDVI will positively affect food consumption via food supplies. (30) found that decreasing NPP would lead to degradation, which affects household decision about livestock breeding and further affect the household food production in grassland. Other studies on Niger and Yemen also found that the decrease of NPP negatively affected the local food supply (45). To summarize, the spatial heterogeneity in ecological resources would subsequently lead to the changes observed in food supplies (mainly meat and dairy products) among these grassland areas, which in turn caused spatial heterogeneity in their food consumption and nutritional structures.

**Table 9:** Relationships between NPP and food consumption among grassland transects.

	**Grains**	**Vegetables**	**Fruits**	**Mutton**	**Beef**	**Chicken**	**Pork**	**Fish**	**Milk**	**Eggs**	**Alcohol**	**Tea**	**Bean**	**Oil**	**Sugar**
**NPP**															
Hulun Buir	−0.228	−0.277	0.058^*^	0.021^*^	0.028^*^	0.016	0.037	0.012^**^	0.055	0.024^*^	0.099^**^	−0.031^*^	0.010	0.050^**^	0.000
	(0.193)	(0.143)	(0.021)	(0.008)	(0.010)	(0.008)	(0.020)	(0.003)	(0.038)	(0.008)	(0.027)	(0.011)	(0.029)	(0.016)	(0.001)
Xilingol	0.048	−0.003	0.062^*^	−0.022	0.013	0.011^*^	−0.028	0.001	0.103	0.017^**^	0.018	−0.018	−0.016	0.008	−0.006
	(0.296)	(0.117)	(0.025)	(0.053)	(0.024)	(0.004)	(0.048)	(0.002)	(0.062)	(0.004)	(0.018)	(0.101)	(0.016)	(0.011)	(0.003)
Ordos	0.743	0.357^*^	0.209	0.096	0.044	0.049^*^	0.185^*^	0.006^*^	0.067	0.046^*^	−0.028	0.010	0.023	0.011	−0.022^**^
	(0.645)	(0.116)	(0.181)	(0.089)	(0.028)	(0.019)	(0.069)	(0.003)	(0.040)	(0.015)	(0.023)	(0.013)	(0.015)	(0.027)	(0.005)

*Sources: calculated by authors, regression with Tobit model*.

* *p < 0.05*;

** *p < 0.01*.

The nuanced differences among these ecosystems may affect regional food supplies. Similar findings were also reported in previous studies. A study conducted in Tibetan Plateau, China among 1017 rural residents found that limited food supply in plateau areas affected local rural residents' diet patterns, especially in the herder group (42). Another study among rural residents in Inner Mongolia, China showed that the traditional diet pattern was characterized by high intakes of starch and sugar, pork, pickled vegetables/dried vegetables and corns, and the local geographic factors limited residents' access to fresh vegetables and fruits (41, 44). Meanwhile, our previous study on Inner Mongolia among rural residents in Xilingol showed that local diet pattern had strong local dependence, and spatial changes in diet patterns were also found in Xilingol Grassland transects (44). Moreover, Grebitus et al. (46) found that in the daily food consumption of households, when household production can in itself provide certain food types, residents preferred to consume food obtained by means of their own production. Some studies from other rural areas revealed that local food production affected residents diet patterns (47). Taken together, these research findings suggest that local food supplies have an irreplaceable effect on the food consumption patterns of household residents, especially in rural areas.

### 4.2. Comparison Between Food Consumption and Nutrition Patterns

#### 4.2.1. Food Consumption and Nutrition Patterns of Rural Household Residents in China

**Figure 4:**
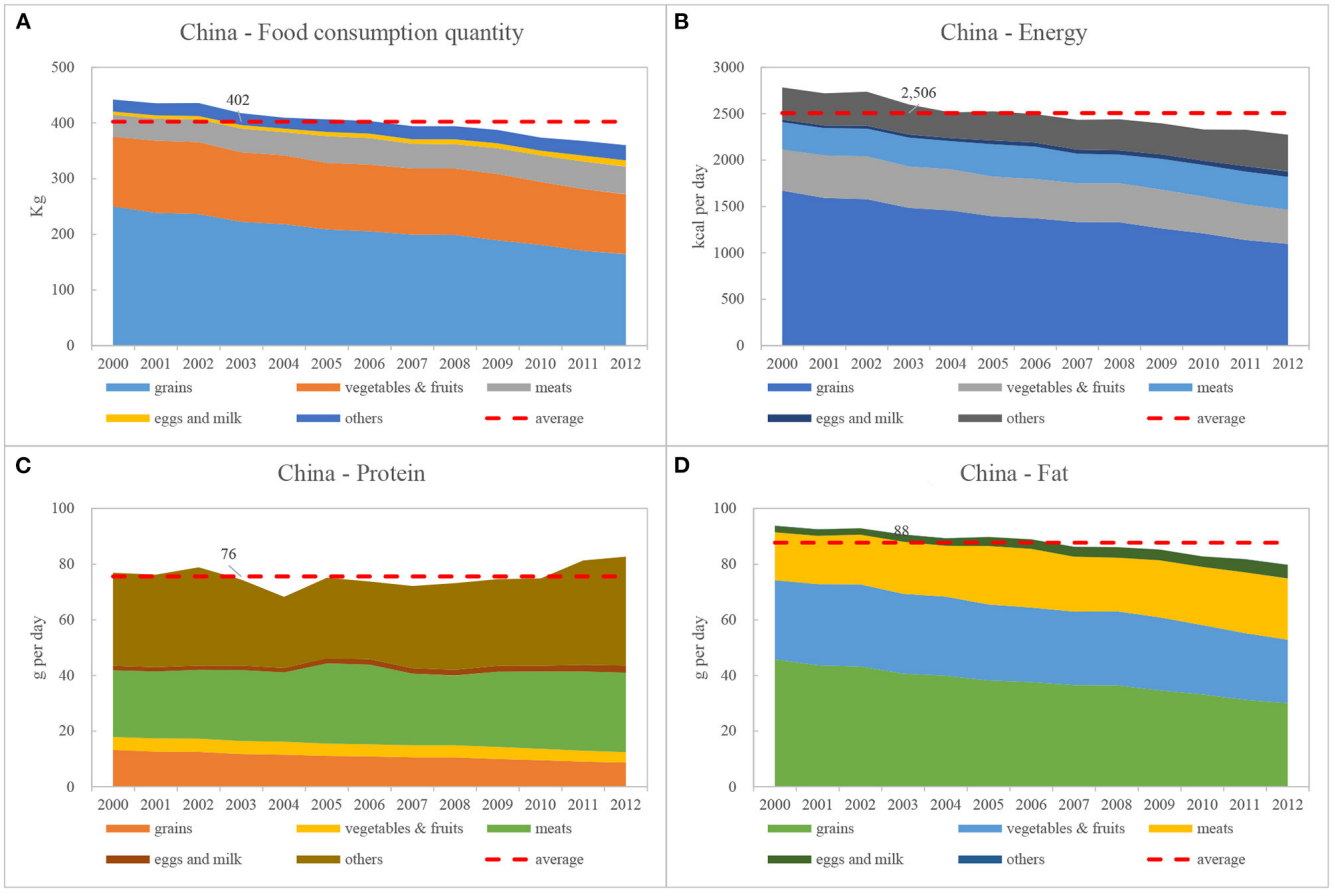
Chinese food consumption and nutritional structure between 2000 and 2012. Data source: National Bureau of Statistics of China. Arranged and calculated by the authors of this study. **(A)** China—Food consumption quantity, **(B)** China—Energy, **(C)** China—Protein, **(D)** China—Fat.

Compared to the food consumption of rural household residents throughout China, the total food consumption quantity of household residents in these three Inner Mongolian grassland areas was less than the national average (i.e., the quantity of total food consumed was <402 kg; Figures 2, 4). From a structural point of view, grains, vegetables and fruits, meat (except for Ordos), and “others” (except for Hulun Buir) were all lower than the national average. Only egg and milk consumption (mainly due to the higher consumption of milk and dairy products) was generally higher than the national average (Figures 2, 4).

Especially from the perspective of meat consumption, rural household residents of Inner Mongolian (i.e., those that reside in pastoral areas) consume a relatively high proportion of beef and mutton (especially in Xilin Gol and Ordos), although they consume almost no meat and poultry products (MPP). For China as a whole, the main sub-categories of meat consumed daily by rural residents are MPP and pork (Tables 6, 10).

**Table 10:** Meat consumption quantity of Chinese rural residents (average values from 2000 to 2012) (kg).

**Food**	**Mean**	**Variance**	**Max**	**Min**
Mutton	0.78	0.03	0.90	0.60
Beef	0.65	0.05	0.50	0.50
Chicken	3.68	0.18	4.50	2.80
Pork	14.00	0.24	12.70	2.90
Fish	4.88	0.14	5.40	3.90
Meat and poultry products	20.75	0.52	23.50	18.20

*Data source: National Bureau of Statistics of China*.

The food nutrition composition of rural household residents of Inner Mongolia, which corresponds to the quantity of food consumed, also showed regional characteristics, which differed from national averages (Figures 2, 4). Although grains and meat are the main sources of energy supplies for household residents of Inner Mongolia, county-level values are relatively balanced.

Grain is the main source of protein supplies for residents of Inner Mongolia, while meat and “others” are the main sources nationally, followed by grains (Figures 2, 4). This suggests that protein sources for rural household residents of Inner Mongolia must be further optimized and improved.

Meat is the most important source of fat supplies for household residents of Inner Mongolia, while vegetables, fruits, and meat are the main sources nationally, the latter being more balanced and therefore healthier (Figures 2, 4).

From the perspective of protein supply, for the residents of Inner Mongolia, grain is the main source of protein supply, while at the national level, meat and others are the main source of supply, followed by grain (Figures 2, 4). It shows that the sources of protein in the food intake of the rural residents of the Inner Mongolia needs to be further optimized and improved;

From the perspective of fat supply, the meat of the residents of Inner Mongolia is the most important source of fat supply, while at the national level, the three categories of food, vegetables and fruits, and meat provide the residents with fat in a balanced manner, which is relatively more balanced and healthier (Figures 2, 4).

#### 4.2.2. Food Consumption and Nutrition Among Countries With Similar Steppe Ecoregions

This study compared food consumption and nutritional structure patterns of rural household residents in three representative grassland areas of Inner Mongolia with corresponding grassland land-use types from several other countries. Food consumption patterns of household residents were obtained from the United Kingdom (UK), New Zealand, Mongolia, Kazakhstan, and Argentina and were compared to corresponding indicators of household residents from the three Inner Mongolian grassland areas selected for this study.

**Figure 5:**
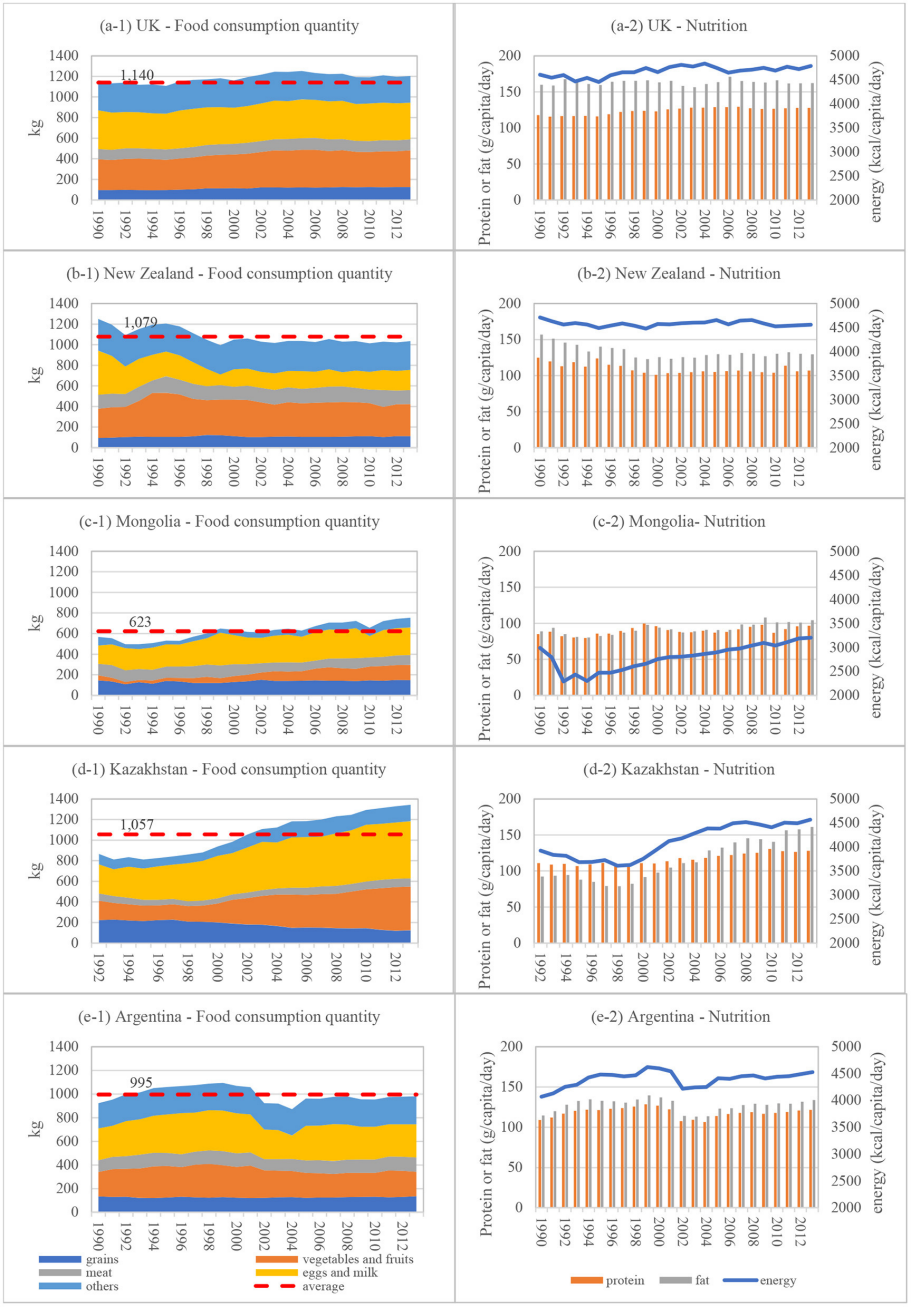
Food consumption and nutritional structure of residents in other typical grassland countries or regions. Data source: FAO database (http://www.fao.org/faostat/en/#data). **(A-1)** UK—food consumption quantity, **(A-2)** UK—nutrition, **(B-1)** New Zealand—food consumption quantity, **(B-2)** New Zealand—nutrition, **(C-1)** Mongolia—food consumption quantity, **(C-2)** Mongolia—nutrition, **(D-1)** Kazakhstan—food consumption quantity, **(D-2)** Kazakhstan—nutrition, **(E-1)** Argentina—food consumption quantity, **(E-2)** Argentina—Nutrition.

By comparing results (as shown in Figures 2, 3, 5), we found that the food consumption patterns of household residents of Inner Mongolia were less than those of other grassland areas in other countries. From a structural perspective, the proportion of food consumed was relatively high, which could have resulted from national consumption behavior patterns that may in turn have influenced the regional behavior of household residents.

Surprisingly, the proportion of protein consumed by rural residents of Inner Mongolia was higher than the national average but less than the proportions of eggs and milk.

Additionally, the consumption of “others” was also relatively high in economically developed countries, such as the UK, New Zealand, and Argentina, while the consumption of “others” in Inner Mongolia exhibited similar characteristics to Mongolia and Kazakhstan.

Furthermore, nutritional characteristics of economically developed countries such as the UK, New Zealand, and Argentina were high in calories, fat, and proteins (Figure 5). Generally, residents of the Inner Mongolia subsists largely on a low-calorie, high-protein, and low-calorie fat-based diet (Figure 3), and overall structural characteristics are similar to those of Mongolia (Figure 5).

### 4.3. Implications for Improving the Food and Nutritional Structures of Pastoral Areas

Our results and related discussion have shown that two critical impact factors affected the consumption structure of household residents in grassland areas of Inner Mongolia: (a) food supplies (ecological factors), and (b) regional socioeconomic status. However, the two factors show different effects. Ecological factors typically have an immediate effect on the daily food consumption of residents via the food supply chain (30, 48). The positive relationship between local food produce and diet was widely analyzed in previous studies (47, 49). Specifically, in this study household residents consume what they harvest, such as milk, mutton, or beef. Meanwhile, socioeconomic factors have a long-term effect on household diet patterns. Previous studies showed that socioeconomic status would affect household food consumption behaviors and change household diet patterns potentially (4, 50, 51). In our study, we found the trends that under socioeconomic development, the food consumption patterns of household residents from underdeveloped countries would shift to the developed countries' ones. Moreover, the latter food consumption patterns were more balanced and stable for all food categories as well as being high in energy, proteins, and fat. However, the change in food consumption patterns was usually with a long time (52).

Our findings provide a reference from which to explore both the potential and direction for improving food consumption structures and patterns of rural household residents of Inner Mongolia. However, it should be noted that Chinese people tend to consume a greater amount of plant-based food products compared to other countries (Figures 4, 5), which in itself is relatively environmentally friendly. On the other hand, this type of food system is more vulnerable, and it lacks reliance when faced with crises, such as the COVID-19 pandemic or natural disasters (2).

In actual fact, the COVID-19 pandemic had an effect on the food supply chain of China and, consequently, local household residents as early as January–March 2020 (53). Thus, it is necessary for both local household residents and local governments to face challenges in improving the reliance on their local food systems. However, Inner Mongolian food consumption structures and patterns are unstable and undergo unpredictable changes along with socioeconomic development. The Government of China and other stakeholders and policymakers should therefore increase their effort in adapting regional food structures and guide local people in raising their awareness to construct a healthy food consumption framework and to reduce the overconsumption of food or food waste into the future, which will greatly benefit the health of local residents as well as their local environments.

### 4.4. Limitations of Our Study

The food consumption quantity of residents of typical grassland areas in other countries is higher than that of Inner Mongolia (data obtained from the Food and Agriculture Organization [FAO] of the United Nations [UN]), and this could be the result of different statistical calibration approaches. However, our study still revealed differences among the grassland areas investigated in Inner Mongolia, and our results also provided evidence for nuanced variation in the regional differences in food consumption patterns of grassland areas. Moreover, although food consumption data from grassland areas revealed interannual fluctuations due to adjustments in statistical calibrations, this does not alter real-world conditions and still reveals similar trends in local food consumption patterns.

Owing to issues related to data availability, we were only able to obtain food consumption data from the Inner Mongolia. Moreover, only the statistical data and data from open-access platforms were used in this study and the data sources are with different adjusting methods. These may affect the fidelity of this study. However, our study was able to reveal food consumption, nutritional structures, and resultant changes in three typical grassland areas of Inner Mongolia. Additionally, our results provide evidence for food consumption pattern changes of local household residents along with livelihood changes, namely, from nomadism to set stocking. The changes and challenges revealed in this study are intended to promote deliberation on the latest issues related to regional sustainable development.

## 5. Conclusions

The spatial heterogeneity of rural household food consumption and nutritional patterns in grassland transects were revealed in this study from a multidisciplinary perspective. Our analysis implies that in grassland areas like Inner Mongolia, food supply was one of the key impact factors for food consumption and nutritional patterns, which are directly affected by local land use and land productivity; while socioeconomic status would affect residents' food consumption patterns in the long term. This study is intended to provide a reference for policymakers and stakeholders to develop sustainable development policies.

## Data Availability Statement

The original contributions presented in the study are included in the article/supplementary material, further inquiries can be directed to the corresponding author/s.

## Author Contributions

WY and HJ conceived and designed the methods and framework. WY analyzed the data, wrote the manuscript, contributed to the interpretation of the data, discussion of results, and writing of the manuscript. CW and HW contributed to the data collection and data analysis. CS contributed to the data collection, interpretation of the data, and discussion of results. All authors read and approved the final manuscript.

## Funding

This research was supported by National Natural Sciences Foundation of China (No. 71673091), National Postdoctoral Science Foundation of China (No. 2021M690200), the National Social Science Fund of China (20CGL063), and the Natural Sciences Foundation of China (No. M-0342).

## Conflict of Interest

The authors declare that the research was conducted in the absence of any commercial or financial relationships that could be construed as a potential conflict of interest.

## Publisher's Note

All claims expressed in this article are solely those of the authors and do not necessarily represent those of their affiliated organizations, or those of the publisher, the editors and the reviewers. Any product that may be evaluated in this article, or claim that may be made by its manufacturer, is not guaranteed or endorsed by the publisher.
